# Colicin E3 cleavage of 16S rRNA impairs decoding and accelerates tRNA translocation on *Escherichia coli* ribosomes

**DOI:** 10.1111/j.1365-2958.2008.06283.x

**Published:** 2008-06-18

**Authors:** Lorna E Lancaster, Andreas Savelsbergh, Colin Kleanthous, Wolfgang Wintermeyer, Marina V Rodnina

**Affiliations:** 1 Institute of Molecular Biology, University of Witten/Herdecke58448 Witten, Germany; 2 Department of Biology, University of YorkYork YO10 5YW, UK; 3 NIBSC, Blanche Lane, Potters BarHertfordshire, EN6 3QG, UK; 4 Institute of Physical Biochemistry, University of Witten/Herdecke58448 Witten, Germany; 5 Max Planck Institute of Biophysical Chemistry, Department of Physical Biochemistry37077 Göttingen, Germany

## Abstract

The cytotoxin colicin E3 targets the 30S subunit of bacterial ribosomes and specifically cleaves 16S rRNA at the decoding centre, thereby inhibiting translation. Although the cleavage site is well known, it is not clear which step of translation is inhibited. We studied the effects of colicin E3 cleavage on ribosome functions by analysing individual steps of protein synthesis. We find that the cleavage affects predominantly the elongation step. The inhibitory effect of colicin E3 cleavage originates from the accumulation of sequential impaired decoding events, each of which results in low occupancy of the A site and, consequently, decreasing yield of elongating peptide. The accumulation leads to an almost complete halt of translation after reading of a few codons. The cleavage of 16S rRNA does not impair monitoring of codon–anticodon complexes or GTPase activation during elongation-factor Tu-dependent binding of aminoacyl-tRNA, but decreases the stability of the codon–recognition complex and slows down aminoacyl-tRNA accommodation in the A site. The tRNA–mRNA translocation is faster on colicin E3-cleaved than on intact ribosomes and is less sensitive to inhibition by the antibiotic viomycin.

## Introduction

During times of stress, some strains of *Escherichia coli* produce the plasmid-encoded cytotoxin colicin E3. It is a ∼60 kDa protein with three domains that are required for the toxin to enter and kill the cell. The receptor binding domain binds to the outer membrane protein, BtuB, of competing *E. coli* (Imajoh *et al*., 1982), and the translocation domain then interacts with the Tol complex of proteins involved in the maintenance of outer membrane stability (Garinot-Schneider *et al*., 1997; Bouveret *et al*., 1998). The cytotoxic domain then interacts directly with the bacterial inner membrane and is finally transported to the cytoplasm, possibly through the action of FtsH (Mosbahi *et al*., 2004; Walker *et al*., 2007). To prevent the producing cell from committing suicide, an inhibitor protein known as the immunity protein Im3 is expressed with the toxin. Im3 forms a complex with the cytotoxic domain of colicin E3 with a binding affinity of 10^−12^ M and inactivates the RNase (Jakes and Zinder, 1974a; Kleanthous and Walker, 2001; Walker *et al*., 2003).

Once inside the cell, the E3 rRNase (cytotoxic) domain specifically cleaves the phosphodiester bond between A1493 and G1494 of 16S rRNA of the small ribosomal subunit (30S) and abolishes protein synthesis (Senior and Holland, 1971). The cleavage site is situated in the decoding site (A site) of the ribosome in helix 44 of 16S rRNA and is adjacent to two universally conserved adenine residues, 1492 and 1493, which are involved in cognate tRNA selection. In recent years, the colicin E3 rRNase domain has been isolated and purified, and its structure and function studied in detail. The crystal structure of the rRNase domain of colicin E3 in complex with Im3 was solved (Carr *et al*., 2000), providing insight into its function. The interaction between E3 and Im3 was also studied biochemically (Walker *et al*., 2003) and mutational analysis has identified residues which are presumed to take part in rRNase activity (Walker *et al*., 2004). The reaction requires the catalytic triad of histidine, glutamic acid and aspartic acid, which all act in general acid-base catalysis of the hydrolysis of the A1493–G1494 phosphodiester bond. Im3 was found to bind the rRNase domain at a position distant from the active site (‘exosite’). The active site of colicin E3 is not blocked by the immunity protein; rather, it is believed that Im3 inhibits the E3 rRNase by steric and electrostatic occlusion, thereby abolishing colicin E3 rRNase binding to the ribosome.

Previous studies of the mechanism of colicin E3 action on the ribosome focused on the structure of ribosomes, experimental conditions required for cleavage to occur and the specific cleavage site (Boon, 1972; Bowman, 1972; Dahlberg *et al*., 1973; Kaufmann and Zamir, 1973; 1975; Ohno-Iwashita and Imahori, 1977; Jakes and Zinder, 1974b). Very few investigations into the stage of translational arrest have been performed. Cloacin DF13, which targets *Enterobacter cloacae* and has a very similar mode of action as colicin E3, has been studied in more detail. It was reported that cloacin DF13-treated ribosomes were able to form initiation complexes, but the function of translation initiation factor 1 (IF1) was altered because it could no longer increase the rate of exchange between ribosome subunits or the rate and extent of IF3-mediated dissociation of 70S ribosomes (Baan *et al*., 1978b). Although polypeptide chain elongation could take place once the 16S rRNA is cleaved, the binding of the aminoacyl-tRNA (aa-tRNA) to the A site of the ribosome was reduced (Baan *et al*., 1978a). The cleaved ribosomes exhibited slightly increased translational fidelity and produced polypeptides that were shorter than those on control ribosomes, although the length of the peptides was not estimated (Twilt *et al*., 1979). In the case of colicin E3, it is not clear whether the initiation complex can be formed once the cleavage has taken place. It was reported that colicin E3-treated ribosomes were unable to form initiation complexes (Tai and Davis, 1974; Tai, 1975), whereas the initiation complex was formed when natural mRNA was used (Kaufmann and Zamir, 1975). The effect of colicin E3 on other steps of translation was not studied.

Here we examine which elemental steps of translation are affected by colicin E3 cleavage, using a reconstituted *in vitro* system in which each step of translation can be followed in real time by rapid kinetics. Our data suggest that the inhibitory effect of colicin E3 originates from impaired decoding. In contrast, tRNA–mRNA translocation through the ribosome becomes faster upon cleavage, and other steps, including initiation and peptide bond formation, are not significantly affected.

## Results

### Cleavage of 16S rRNA by colicin E3

**Fig. 1:**
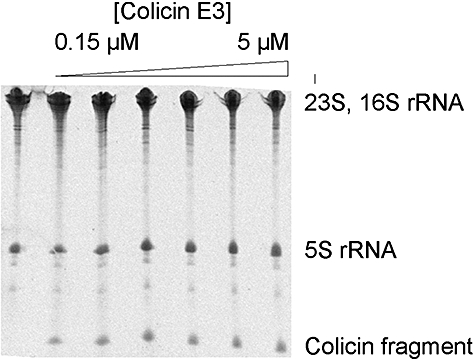
Cleavage of rRNA by colicin E3. Ribosomes (1 μM) were treated with increasing concentrations of colicin E3, and rRNA fragments analysed on denaturing polyacrylamide gel electrophoresis.

To test the extent and the specificity of 16S rRNA cleavage by colicin E3, 70S ribosomes were incubated for 30 min at room temperature with different concentrations of colicin E3, and the rRNA was extracted and analysed by denaturing gel electrophoresis (see *Experimental procedures*) (Fig. 1). The treatment resulted in an rRNA fragment of 50 nucleotides, which did not appear in the absence of colicin E3 (lane 1). The appearance of the fragment was observed even at the lowest concentration of colicin E3 tested (Fig. 1); according to RT-PCR, cleavage of 16S rRNA was nearly complete (95%, data not shown). The cleavage was specific, as indicated by the distinct bands of the colicin E3 fragment, 5S rRNA and 16/23S rRNA. The same results were obtained when 70S initiation complexes carrying mRNA and [^3^H]fMet-tRNA^fMet^ in the P site were used (data not shown). In all following experiments, equimolar concentrations of colicin E3 and 70S ribosomes were used (as in lane 5, Fig. 1). Unless indicated otherwise, after colicin E3 treatment, Im3 was added to the reaction mixture to remove colicin E3 from the ribosome. This colicin E3–Im3 interaction is very strong with picomolar affinity (Walker *et al*., 2003), and Im3 completely removes colicin E3 rRNase from the ribosome, as measured with fluorescence-labelled colicin E3 (Lancaster, 2005).

### Overall translation

**Fig. 2:**
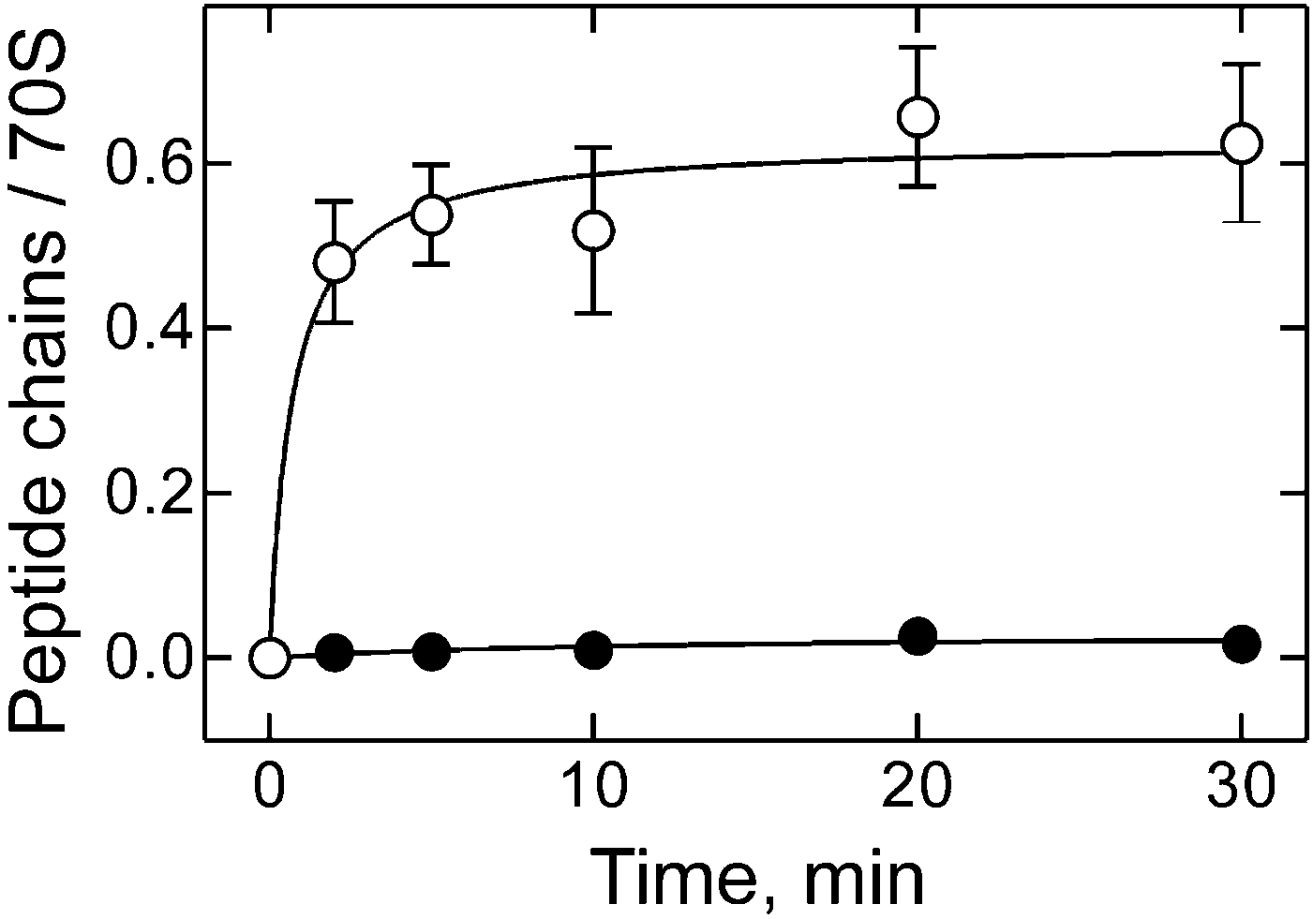
Inhibition of *in vitro* translation by colicin E3. Ribosomes treated with colicin E3 (closed circles) or control ribosomes (open circles) were used in a translation system reconstituted of purified initiation and elongation factors, f[^3^H]Met-tRNA^fMet^, Lep75 mRNA and a mixture of aa-tRNAs containing [^14^C]Leu-tRNA. The fraction of ribosomes carrying a nascent peptide was calculated from the amount of f[^3^H]Met in the peptide. In all cases where translation has taken place, full-length peptides were synthesized, as indicated by the f[^3^H]Met/[^14^C]Leu ratio (data not shown).

Colicin E3-treated ribosomes were tested in a translation assay reconstituted from purified components (Buskiewicz *et al*., 2004) (Fig. 2), using a truncated mRNA coding for the first 75 amino acids of leader peptidase (Lep). The mRNA construct does not contain a stop codon at the end of the mRNA and termination of translation does not take place. Therefore, any effects of colicin E3 on translation would imply initiation or elongation of translation as targets for inhibition. Ribosomes treated with colicin E3 showed no detectable translation activity, while control ribosomes produced the expected amount of protein (maximum one peptide chain per ribosomes can be obtained), indicating that one or more elemental steps during initiation or elongation of translation are impaired by colicin E3 treatment. The complete inhibition of translation is consistent with practically complete cleavage of 16S rRNA indicated by RT-PCR analysis (data not shown).

### Initiation

**Fig. 3:**
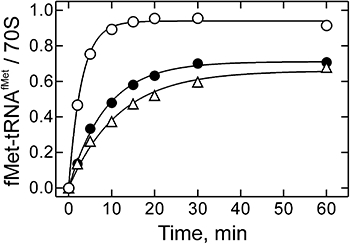
Initiation of translation. Time-course of f[^3^H]Met-tRNA^fMet^ (0.3 μM) binding to 70S ribosomes (0.2 μM) treated with E3 alone (open triangles), with E3 and Im3 (closed circles), and control ribosomes (open circles) in the presence of mRNA (0.3 μM), initiation factors (0.3 μM each), GTP (1 mM) in buffer A at 37°C.

To identify the step that is impaired by the cleavage of 16S rRNA, colicin E3-treated ribosomes were tested for their ability to perform factor-dependent initiation (Fig. 3) (Calogero *et al*., 1988; Tomsic *et al*., 2000). Initiation with colicin E3-treated ribosomes was somewhat slower and less efficient, reaching ∼75% compared with control ribosomes. The effect was observed regardless whether colicin E3 was removed from the ribosomes by adding the immunity protein Im3 or not. However, the effect was moderate and cannot account for the complete block of translation after colicin E3 cleavage (Fig. 2), indicating that other steps of translation must be affected.

### Decoding

**Fig. 4:**
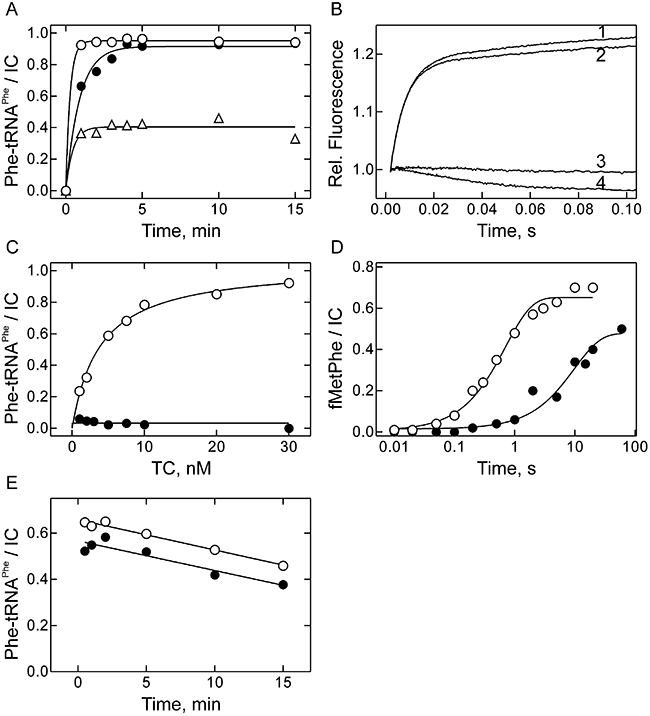
Decoding. A. Time-course of binding of EF-Tu·GTP·[^14^C]Phe-tRNA^Phe^ to the A site of initiation complexes prepared from ribosomes treated with colicin E3 alone (open triangles), with colicin E3 and Im3 (closed circles), and control ribosomes (open circles). The amount of [^14^C]Phe-tRNA^Phe^ bound was normalized to the amount of f[^3^H]Met-tRNA^fMet^ in the 70S initiation complex (IC). B. Time-course of Pi release from EF-Tu. Initiation complexes from control (curves 1 and 3) and colicin E3-cleaved (2 and 4) ribosomes were mixed rapidly with EF-Tu·GTP·Phe-tRNA^Phe^ (1 and 2) or buffer (3 and 4). Pi release was measured by the fluorescence change of MDCC-phosphate-binding protein (PBP) upon Pi binding. C. Codon–recognition complex formation. 70S initiation complexes (2 nM) were mixed with increasing concentrations of EF-Tu(H84A)·GTP·[^14^C]Phe-tRNA^Phe^. Ribosomes were treated with colicin E3 and Im3 (closed circles) or Im3 only (open circles). D. Time-course of peptide bond formation upon addition of EF-Tu·GTP·[^14^C]Phe-tRNA^Phe^ (1.2 μM) to initiation complexes (0.4 μM). Same symbols as above. E. Stability of fMetPhe-tRNA^Phe^ binding to the A site. Initiated ribosomes (0.2 μM) were mixed with EF-Tu·GTP·[^14^C]Phe-tRNA^Phe^ (0.15 μM) and the time-course of dissociation measured (Semenkov *et al*., 2000). Same symbols as above.

The elongation step of protein synthesis comprises decoding of the mRNA codon in the A site, peptide bond formation and tRNA–mRNA translocation from the A site to the P site. To test whether the E3-promoted cleavage and/or the presence of colicin E3 in the A site affect tRNA binding, ribosomes were incubated with ternary complexes consisting of elongation-factor Tu (EF-Tu)·GTP·[^14^C]Phe-tRNA^Phe^, and the amount of [^14^C]Phe-tRNA^Phe^ bound to the A site was determined by nitrocellulose filtration (Rodnina *et al*., 1995) (Fig. 4A). Control ribosomes bound [^14^C]Phe-tRNA^Phe^ rapidly with more than 90% efficiency. Ribosomes treated with colicin E3 followed by removal of the RNase with the immunity protein Im3 showed equally efficient A-site binding, although at a reduced rate. When colicin E3 was not removed from the ribosomes by immunity protein Im3, not only the rate of A-site binding was strongly affected, but the binding was also much less efficient, reaching a plateau at about 40%. This effect is in agreement with the notion that colicin E3 and [^14^C]Phe-tRNA^Phe^ compete for the A site (Kaufmann and Zamir, 1973). For further experiments, only ribosomes treated with colicin E3 followed by removal of E3 with immunity protein Im3 were used.

The EF-Tu-dependent A-site binding of aa-tRNA involves several elemental steps, such as docking of the ternary complex EF-Tu·GTP·aa-tRNA to the ribosome, formation of the codon–recognition complex, GTP hydrolysis by EF-Tu and accommodation of aa-tRNA in the A site (Rodnina *et al*., 2005). GTP hydrolysis – as measured using an indicator reaction monitoring free Pi released from EF-Tu – is not inhibited by colicin E3 cleavage of 16S rRNA (Fig. 4B). Given that GTP hydrolysis is equally efficient on colicin E3-treated and control ribosomes, the preceding steps, i.e. binding of the ternary complex to the ribosome and codon recognition, are also not impaired appreciably. However, this does not exclude that the affinity of ternary complex binding to the ribosome may be altered. This was tested using the EF-Tu mutant EF-Tu(H84A) which is deficient in GTP hydrolysis (Daviter *et al*., 2003). Because GTP hydrolysis is blocked, ternary complex binding is reversible and – provided that the complex does not dissociate during nitrocellulose filtration – the equilibrium dissociation constant (*K*_d_) of the codon–recognition complex can be measured. Titration of 70S initiation complexes with EF-Tu(H84A)·GTP·[^14^C]Phe-tRNA^Phe^ gives *K*_d_ = 3 ± 0.4 nM for control ribosomes, consistent with previous measurements (Daviter *et al*., 2003). In contrast, no significant binding of ternary complex to initiation complexes containing colicin E3-treated ribosomes can be detected within the concentration range tested (Fig. 4C), indicating that the stability of the codon–recognition complex is strongly decreased upon colicin E3 cleavage. It should be noted that nitrocellulose filtration is a non-equilibrium method, implying that unstable complexes may dissociate upon washing of the filters.

Another effect on A-site binding was observed when the rate of fMetPhe dipeptide formation was monitored by quench-flow (Fig. 4D). Colicin E3-treated ribosomes were 15-fold slower in dipeptide formation (0.1 s^−1^ compared with 1.5 s^−1^ on control ribosomes), and the yield of dipeptide was reduced to about 70% compared with control ribosomes. It is unlikely that the peptidyl transfer reaction *per se* was affected by the cleavage of 16S rRNA, because the peptidyl transferase centre is located on the 50S subunit and the rate of fMet-tRNA^fMet^ reaction with the aa-tRNA analogue puromycin (Pmn) was not altered (about 0.5 s^−1^; data not shown). The decreased rate of fMetPhe formation is therefore likely to be caused by a decreased rate of Phe-tRNA accommodation in the A site, whereas the lower final level indicated drop-off of part of the Phe-tRNA^Phe^ from the ribosome during the accommodation phase.

Once [^14^C]Phe-tRNA^Phe^ was accommodated and peptide bond formation had taken place, the stability of the fMetPhe-tRNA^Phe^ binding was similar on colicin E3-treated and control ribosomes (Fig. 4E). This was tested by the addition of a slight excess of initiation complexes to ternary complexes to ensure full binding of the ternary complex and fMetPhe-tRNA formation. The resulting complex was incubated at 37°C and samples taken at different time points were analysed by nitrocellulose filtration. Control and colicin E3-treated ribosomes showed the same slow loss of fMet[^14^C]Phe-tRNA^Phe^ from the A site (Peske *et al*., 2004), i.e. E3 cleavage did not destabilize peptidyl-tRNA binding in the A site.

### Translocation

**Fig. 5:**
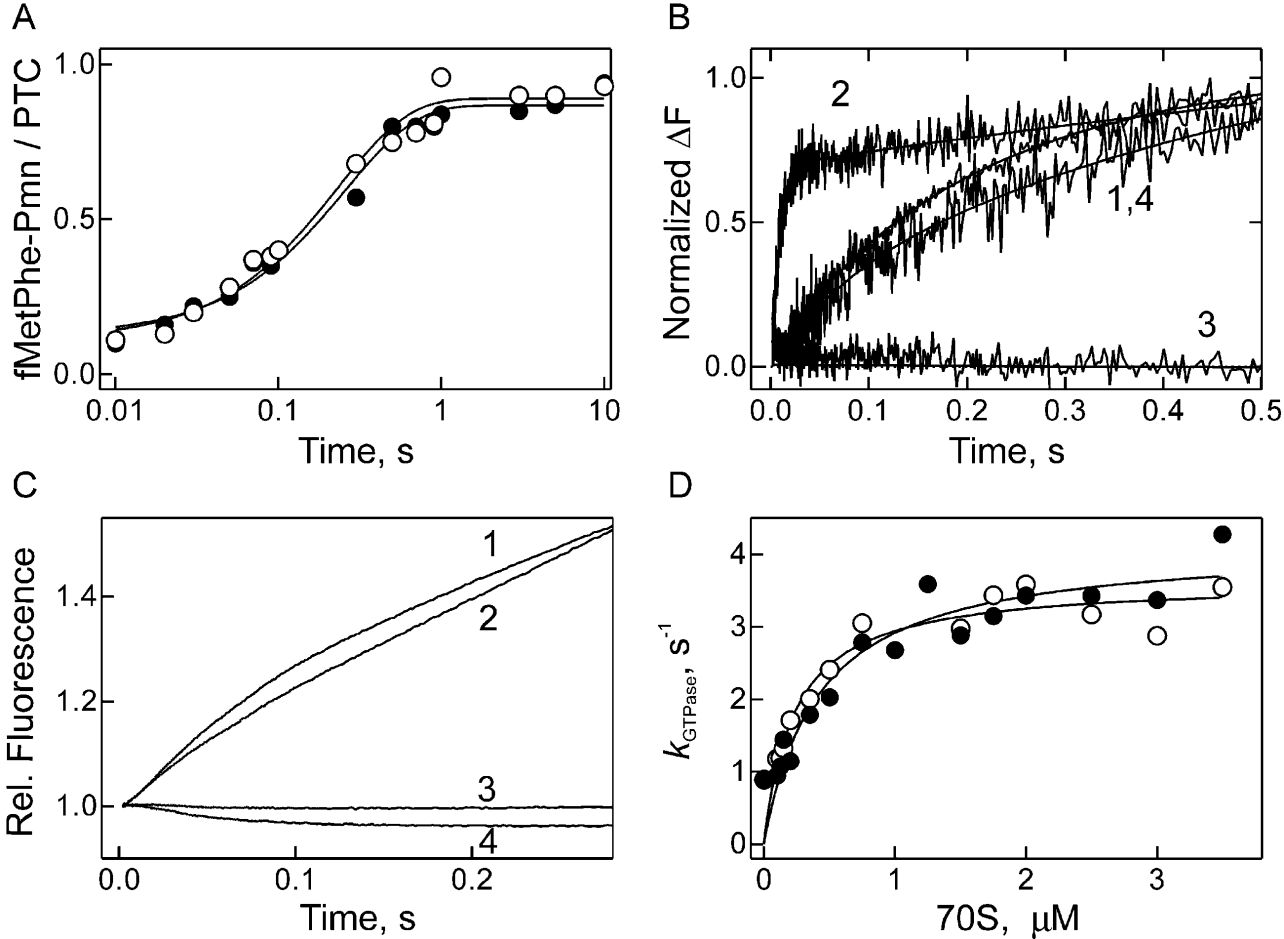
Translocation. A. Colicin E3-cleaved (closed circles) or control (open circles) pre-translocation complexes (0.1 μM) were mixed in a quench-flow apparatus with EF-G·GTP (3 μM) and Pmn (10 mM) and translocation analysed by formation of fMetPhe-Pmn. The amount of pre-translocation complex (PTC) was determined by HPLC analysis of fMetPhe. B. Translocation was monitored by the fluorescence change of fMetPhe-tRNA^Phe^(Prf16/17). Curve 1 – control ribosomes; curve 2 – colicin E3-cleaved ribosomes; curve 3 – control ribosomes in the presence of viomycin (200 μM); curve 4 – colicin E3-cleaved ribosomes with viomycin (200 μM). Normalized ΔF, fluorescence change normalized by the maximum extent of translocation determined by the Pmn reaction. C. Pi release upon GTP hydrolysis by EF-G (curves 1 and 2) or buffer control (curves 3 and 4) with control ribosomes (curves 1 and 3) or colicin E3-cleaved (curves 2 and 4). Pi release was measured by the fluorescence change of MDCC-PBP upon Pi binding (*Experimental procedures*). D. Multiple-turnover GTP hydrolysis by EF-G. Velocity of GTP hydrolysis was measured with catalytic concentrations of EF-G (10 nM) and increasing concentrations of colicin E3-cleaved ribosomes (closed circles) or control ribosomes (open circles) in the presence of excess [γ-^32^P]-GTP (200 μM).

The effect of colicin E3 cleavage on translocation was tested first by quench-flow, monitoring the reaction of peptidyl-tRNA with Pmn (Katunin *et al*., 2002a). Pre-translocation complexes with f[^3^H]Met[^14^C]Phe-tRNA^Phe^ in the A site and deacylated tRNA^fMet^ in the P site were mixed with excess EF-G and Pmn. Peptidyl-tRNA in the A site does not react with Pmn (Fig. 5A). However, as soon as f[^3^H]Met[^14^C]Phe-tRNA^Phe^ is translocated to the P site, the reaction with Pmn takes place rapidly [10 s^−1^ with fMetPhe-tRNA^Phe^ at the present conditions (Katunin *et al*., 2002b)], resulting in the formation of f[^3^H]Met[^14^C]Phe-Pmn, which can be determined by (high-performance liquid chromatography) HPLC. The rate of translocation on control ribosomes was about 10 s^−1^ (see below); therefore, the overall rate of coupled translocation and Pmn reaction is expected to be about 5 s^−1^. It should be noted that a rapid Pmn reaction is indicative of full translocation of tRNAs and mRNA on both ribosomal subunits, because partially translocated peptidyl-tRNA [in the hybrid A/P state (Moazed and Noller, 1989)] reacts with Pmn 500 times more slowly (Sharma *et al*., 2004). The same rate of translocation, 5 ± 1 s^−1^, was found on colicin E3-treated and untreated ribosomes (Fig. 5A). This suggests that the 16S rRNA cleavage does not significantly impair translocation. However, given the limited rate of the indicator Pmn reaction, a moderate stimulation or inhibition of translocation is not excluded by these results.

Rapid kinetics of translocation can be followed by the fluorescence of fluorophores attached either to the tRNA (Robertson and Wintermeyer, 1981; Robertson *et al*., 1986; Rodnina *et al*., 1997; Pan *et al*., 2007) or to the 3′ end of the mRNA (Studer *et al*., 2003; Peske *et al*., 2004). The complexes of colicin E3-treated ribosomes with fluorescence-labelled mRNA were of insufficient stability for kinetic experiments (data not shown). Therefore, single-round translocation was measured using proflavin in fMetPhe-tRNA^Phe^(Prf16/17) as reporter group, and the fluorescence changes during translocation were monitored by fluorescence stopped-flow as described previously (Rodnina *et al*., 1997). On control ribosomes, translocation resulted in a fluorescence increase taking place at a rate of 10 ± 2 s^−1^ (Fig. 5B), as seen in previous experiments (Rodnina *et al*., 1997). The time-course of translocation on colicin E3-cleaved ribosomes showed very fast translocation with a rate of 110 ± 10 s^−1^, implying that translocation is accelerated by the cleavage in 16S rRNA. Furthermore, the antibiotic viomycin, which completely blocks tRNA movement on native ribosomes (Rodnina *et al*., 1997), does not abolish translocation on the ribosomes with the colicin E3 cleavage. In fact, on colicin E3-cleaved ribosomes, the end point of the Pmn reaction was the same (data not shown) in the absence and presence of viomycin, whereas the reaction rate was 4.8 ± 0.1 s^−1^ in the presence of viomycin (Fig. 5B).

To determine whether the GTPase activity of EF-G was affected by colicin E3 cleavage, Pi release from EF-G was measured using a limiting amount of ribosomes and excess EF-G as described (Savelsbergh *et al*., 2003) (Fig. 5C). At these conditions, the first round of Pi release is observed as a burst phase (reflected in a fluorescence increase lasting up to 0.1 s), followed by multiple rounds of GTP hydrolysis, and Pi release (further fluorescence increase). That colicin E3 cleavage had no effect on EF-G turnover was substantiated by measuring the GTPase activity of EF-G using catalytic amounts of factor and excess ribosomes (Fig. 5D). The initial velocities of multiple-turnover GTP hydrolysis were identical on colicin E3-treated and control ribosomes, *k*_cat_ = 4 ± 1 s^−1^.

### Pentapeptide synthesis

**Fig. 6:**
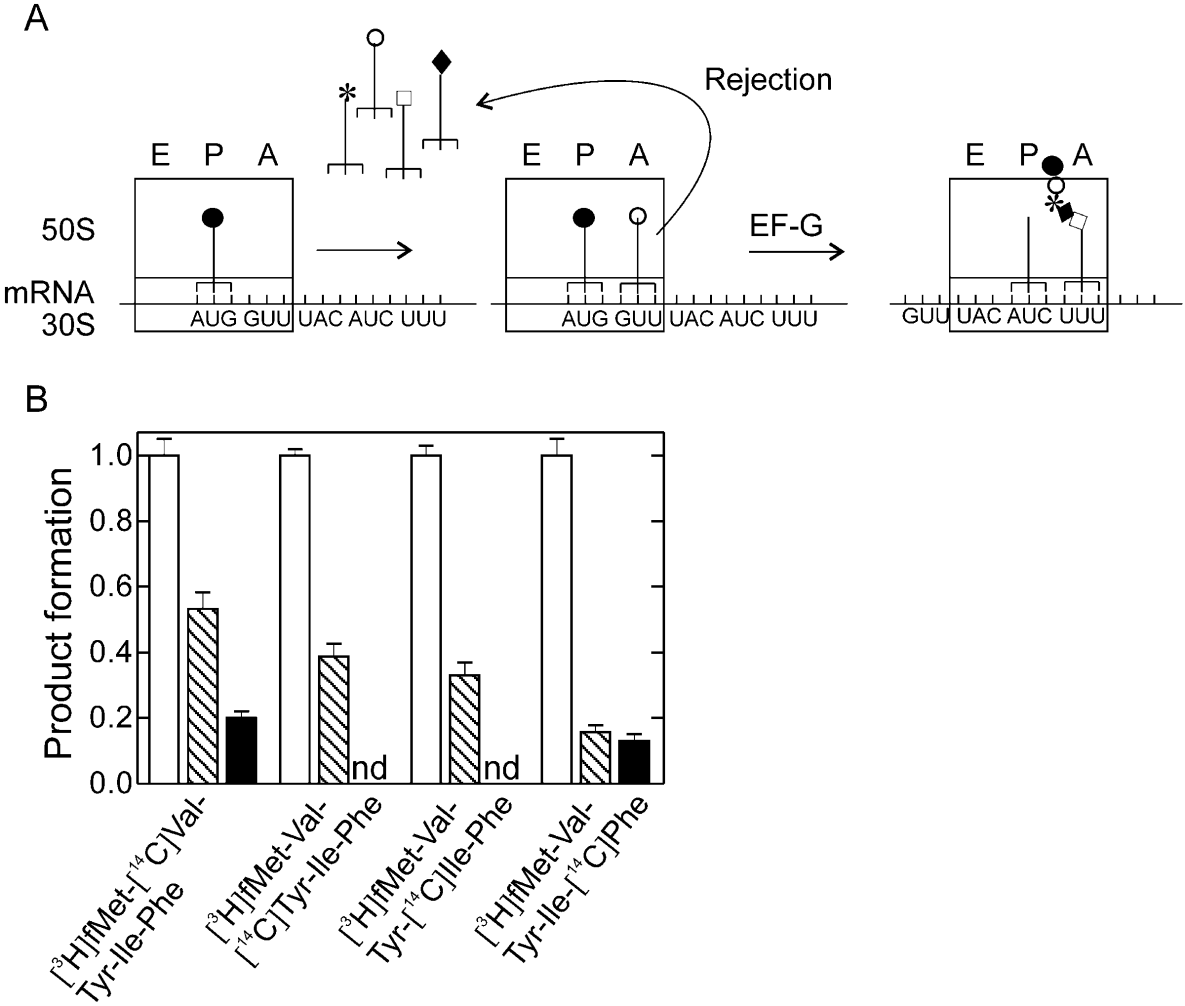
Pentapeptide synthesis. A. Experimental approach. Initiation complexes (1 μM) were mixed with four different purified aa-tRNAs (1.1 μM each) in the presence of EF-Tu (1.65 μM), EF-G (2 μM), GTP (1 mM), phosphoenolpyruvate (3 mM) and pyruvate kinase (0.1 μg μl^−1^). In each cycle, only one aa-tRNA was ^14^C-labelled. B. Binding of aa-tRNA (grey bars, monitored by nitrocellulose filtration) and formation of the pentapeptide (black bars, analysed by HPLC) on colicin E3-cleaved ribosomes, normalized to the respective values obtained with control ribosomes (white bars). About 80% of control ribosomes bound each aa-tRNA and formed the pentapeptide fMetValTyrIlePhe. Labels indicate the position in the peptide of the labelled amino acid being analysed in each row. nd, not determined.

The results shown so far suggest that the strongest effect of colicin E3 cleavage is on A-site binding of ternary complex or aa-tRNA, whereas the effects on initiation, peptide bond formation and translocation are either small or cannot explain the inhibition of translation. To identify the step at which translation is halted, an assay was devised to monitor the stepwise synthesis of a pentapeptide. Initiation complexes with f[^3^H]Met-tRNA^fMet^ in the P site were prepared on an mRNA coding for the pentapeptide fMetValTyrIlePhe (Fig. 6A). In the first sample, initiation complexes were incubated with a 1.1 excess of [^14^C]Val-tRNA^Val^ and unlabelled Tyr-tRNA^Tyr^, Ile-tRNA^Ile^ and Phe-tRNA^Phe^ in the presence of EF-Tu·GTP and EF-G·GTP and the binding of f[^3^H]Met- and [^14^C]Val-tRNA was measured by nitrocellulose filtration. The fraction of ribosomes containing Val-tRNA^Val^ in the A site was determined by the amount of ^14^C radioactivity per initiation complex (Fig. 6B). The results show that [^14^C]Val-tRNA^Val^ binding to the A site was reduced by 50% on colicin E3-cleaved compared with control ribosomes. Because binding of Phe-tRNA to the A site was not appreciably inhibited (Fig. 4A), the reduction of Val-tRNA binding in the presence of the other ternary complexes may indicate that competition between ternary complexes inhibits the reaction, although variations in abilities of individual aa-tRNAs to bind to cleaved ribosomes cannot be excluded. The second sample contained the same components, except that now Tyr was ^14^C-labelled to determine whether the dipeptide was translocated to allow the next aa-tRNA to enter the A site. The experiment was continued for the following two amino acids in the pentapeptide, i.e. with labelled Ile and then Phe (Fig. 6A) to determine if colicin E3-cleaved ribosomes could produce tetra- and pentapeptide, respectively. The results for the binding of the second ternary complex, Tyr-tRNA^Tyr^, show again that translocation is not inhibited by colicin E3 cleavage. If an inhibition of translocation played a role in translation inhibition, then very little binding of Tyr-tRNA^Tyr^ would have been observed. However, 40% of colicin E3-cleaved ribosomes were able to bind Tyr-tRNA^Tyr^ to produce, at least, a tripeptide. In the next steps, even less [^14^C]Ile and [^14^C]Phe were bound to the ribosome, and only about 15 (±5)% of [^14^C]Val or [^14^C]Phe were found in the pentapeptide fraction. By extrapolating these results to longer peptides, one can expect that essentially no peptides longer than about eight amino acid residues will be formed when the ribosomes are cleaved by colicin E3. The progressive decrease in the amount of ribosome-bound aa-tRNA shows that defects in A-site binding, particularly in the presence of competing ternary complexes, is the major reason for the halt of protein synthesis.

## Discussion

### Effect of colicin E3 on translation

Colicin E3 catalyses the cleavage of the phosphodiester bond between nucleotides 1493 and 1494 of 16S rRNA of *E. coli* ribosomes. Cleavage leads to a complete inhibition of protein synthesis in the cell and to cell death. Previous experiments, performed both *in vivo* and in cell lysates, have shown that protein synthesis is abolished when colicin E3 is present (Bowman *et al.*, 1971; Senior and Holland, 1971; Walker *et al*., 2003). Using a translation system reconstituted from purified components for translation initiation and elongation (Buskiewicz *et al*., 2004), we show here that protein synthesis is abolished at the elongation step of translation. The initiation step is affected very little, as it is slighty slower and 30% less efficient with ribosomes treated with colicin E3. Similar effects were observed earlier with ribosomes cleaved by cloacin DF13 and attributed to an impaired function of IF1 (Baan *et al*., 1978b). Inhibition of IF3 or IF2 abolishes initiation on 70S ribosomes; therefore, a much larger effect on initiation would be expected if 16S rRNA cleavage would affect the functions of the latter two factors directly. The known activities of IF1 are: (i) to increase the binding of IF2 and fMet-tRNA^fMet^ to the 30S subunit and (ii) to mediate the function of IF3 in the control of initiation fidelity. In fact, the inhibition of initiation by cloacin DF13 could be overcome by the addition of excess IF3 (Baan *et al*., 1978b), suggesting that the IF1–IF3 interaction was impaired by 16S rRNA cleavage. However, the effects on initiation are too small to account for the observed complete shutdown of protein synthesis. Thus, the overall effect on translation must be due to the inhibition of step(s) during elongation.

The elongation step of protein synthesis entails EF-Tu-dependent binding of aa-tRNA to the A site, peptide bond formation and translocation. Our data show that peptide bond formation and translocation are not inhibited by colicin E3 cleavage (Fig. 5A). The main defect is observed during decoding. When colicin E3 remains bound to the A site in the absence of Im3, the amount of aa-tRNA that binds to the A site is reduced by 60%, probably because of the competition between aa-tRNA and colicin E3 for binding to the decoding centre. When colicin E3 is removed from the ribosomes after the cleavage, the effects are more subtle. The stability of the codon–recognition complex is dramatically reduced and the rate of aa-tRNA accommodation in the A site is decreased ∼200-fold. When a specific ternary complex is present in large excess, colicin E3 cleavage does not prevent aa-tRNA binding and, most, but not all, of initially bound aa-tRNA accommodates properly to participate in peptide bond formation (Fig. 4D). In the presence of a mixture of different ternary complexes, which compete for the A site, the effect of colicin E3 cleavage is more pronounced. However, also in the latter case, the decrease in aa-tRNA binding did not exceed a factor of two in one round of elongation (Fig. 6). However, the accumulation of moderate binding and/or accommodation defects over several rounds of elongation leads to almost complete inhibition in the synthesis of peptides of five amino acids or longer. These results suggest that amino acid incorporation is reduced to zero after the translation of about eight codons. The conclusion is that within the cell, only short peptides would be produced by colicin E3-cleaved ribosomes, leading to cell death.

### Implications for the mechanisms of decoding and translocation

In addition to its importance for bacterial physiology, colicin E3 provides a tool to obtain insight into the dynamic properties of the decoding centre. The colicin E3 cleavage occurs in helix 44 of 16S rRNA next to two adenine residues, 1492 and 1493, which are universally conserved in all organisms. Upon codon recognition by cognate aa-tRNA, A1492 and A1493 change their positions to interact with the minor grove of the first two base pairs of the codon–anticodon complex in a fashion which is specific for Watson–Crick geometry, but does not depend on sequence (Ogle *et al*., 2001). The local conformational changes in the decoding site result in a global rearrangement of the head and shoulder of the 30S subunit, which assume a closed configuration upon formation of the correct codon–anticodon duplex (Ogle and Ramakrishnan, 2005). Most likely, 16S rRNA cleavage between residues 1493 and 1494 does not impair the ability of the ribosome to select cognate codon–anticodon complexes, because the ribosomes with cleaved 16S rRNA were found to be highly accurate in aa-tRNA selection (Twilt *et al*., 1979). Similarly, we observed, by HPLC analysis, that upon synthesis of pentapeptides (Fig. 6), the last amino acid in the sequence (Phe) was incorporated exclusively at the position defined by the respective cognate codon and was not found in dipeptides where it could have been misincorporated at a valine codon (data not shown). This implies that A1492 and A1493 in the cleaved 16S rRNA retain the ability to monitor the codon–anticodon complex. Furthermore, Pi release from EF-Tu was unaffected, indicating that also the preceding steps of ternary complex binding to the ribosome, i.e. codon reading and GTP hydrolysis, proceeded with essentially unchanged rates irrespective of the cleavage. This supports the notion that after colicin E3 cleavage, A1492 and A1493 retain the ability to interact with the codon–anticodon complex, because disruption of the A-minor interactions results in slow GTP hydrolysis (Gromadski *et al*., 2006; Cochella *et al*., 2007). Furthermore, the global closing of the 30S subunit may also be expected to take place, as the restriction of the flexibility within the 30S subunit, e.g. by binding streptomycin (Carter *et al*., 2000), renders GTPase slow even on cognate codons (Gromadski and Rodnina, 2004a).

On the other hand, our data show that colicin E3 cleavage abolishes the stabilization of cognate aa-tRNA in the codon recognition complex, which is observed with intact ribosomes (Ogle *et al*., 2002; Daviter *et al*., 2003; Gromadski and Rodnina, 2004b), or during aa-tRNA accommodation into the A site. Interestingly, the main destabilizing effect was observed with the codon–recognition complex or during aa-tRNA accommodation in the A site. In both complexes, the codon–anticodon interaction comprises the major contact of aa-tRNA to the ribosome, while the 3′ end of the tRNA is either still bound to EF-Tu or moves through the ribosome. Essentially no effect on the stability of peptidyl-tRNA binding to the ribosome was observed at later stages, i.e. after the accommodation of aa-tRNA in the A site, possibly as a result of compensatory tRNA contacts outside the decoding region.

The adverse effects of 16S rRNA cleavage on GTPase activation and the stabilization of the codon–recognition complex imply that the two reactions are not necessarily coupled through rearrangements at the decoding site. This is supported by earlier observations indicating that the binding of paromomycin to the decoding centre, which stabilized the flipped-out conformation of A1492 and 1493 even in the absence of cognate codon–anticodon interaction (Ogle *et al*., 2001), induced rapid GTP hydrolysis in a ternary complex bound to a near-cognate codon (Pape *et al*., 2000). At the same time, the stability of the respective codon–recognition complexes was hardly changed (Pape *et al*., 2000; Ogle *et al*., 2002).

The structural reason for the reduced stability of codon–recognition and proofreading complexes with colicin E3-cleaved ribosomes is unclear. One possible explanation may be a change in the structure of the bridge 2a, which is formed by elements of 16S rRNA (helix 44, positions 1494–1495, 1408–1410) and 23S rRNA (helix 69, positions 1913–1914, 1918)(Yusupov *et al*., 2001), and is one of the contact points between the ribosome and aa-tRNA in the A site (Yusupov *et al*., 2001; Stark *et al*., 2002; Valle *et al*., 2003). If the cleavage between positions 1493 and 1494 changes the structure of the contact site at position 1494, the formation or dynamics of the bridge may be impaired, thus inhibiting the correct interaction with aa-tRNA, which would explain the low stability of the complex. Deletion of helix 69 of 23S rRNA, which destroys the 50S side of bridge 2a, did not impair poly(Phe) synthesis, but increased the fidelity of aa-tRNA selection by a factor of two (Ali *et al*., 2006), which reminds of the fidelity effect of 16S rRNA cleavage (Twilt *et al*., 1979). The effect of helix 69 deletion on aa-tRNA binding during codon recognition and proofreading was not examined (Ali *et al*., 2006).

Another possible explanation for the low stability of the codon–recognition and proofreading complexes involves the notion that the tRNA is mechanically distorted upon decoding (Stark *et al*., 2002; Valle *et al*., 2003; Yarus *et al*., 2003; Daviter *et al*., 2005). In the codon recognition complex, the aa-tRNA undergoes a conformational change (Stark *et al*., 2002; Valle *et al*., 2003), which presumably is coupled to or required for GTPase activation (Rodnina *et al*., 1994a; 1995; Piepenburg *et al*., 2000; Cochella and Green, 2005). The distorted tRNA conformation is stabilized by the contacts of the tRNA with the ribosome and EF-Tu (Stark *et al*., 2002; Valle *et al*., 2003), and conformational rigidity of the decoding centre may be required to maintain the high stability of the complex. The cleavage between positions 1493 and 1494 may render the decoding site less rigid and therefore unable to withstand the strain exercised by the mechanic tension in the tRNA molecule. The resulting relaxation in the tRNA structure may then lead to a loss of contacts with the ribosome, thereby decreasing the stability of the complex. During initial selection of aa-tRNA, weaker binding of cognate aa-tRNA in the A site has little effect on decoding, because the reaction is driven by the rapid forward reactions of GTPase activation and GTP hydrolysis (Rodnina and Wintermeyer, 2001). However, a very low stability of near-cognate complexes, in combination with a low rate of GTPase activation (Gromadski and Rodnina, 2004b), may result in hyperaccurate ribosomes, as has been reported (Twilt *et al*., 1979).

Furthermore, compromised mechanical properties of the decoding site may be responsible for the loss of a portion of aa-tRNA at the proofreading stage, when the 3′ end of aa-tRNA moves from its position on EF-Tu into the peptidyl transferase centre of the ribosome (Gromadski and Rodnina, 2004b). This step requires tight interactions of the tRNA with the ribosome and entails conformational changes of aa-tRNA (Sanbonmatsu *et al*., 2005); increased flexibility of the decoding centre may impair the movement. In fact, aa-tRNA accommodation is slowed down 15 times by colicin E3 cleavage, and a relatively high proportion (30%) of cognate aa-tRNA is lost during proofreading (Fig. 4D). Thus, the cleavage of 16S rRNA between positions 1493 and 1494 does not appear to impair monitoring of codon–anticodon complexes or GTPase activation, but affects the stability of aa-tRNA binding at critical stages of decoding and its ability to move within the ribosome, probably by increasing the flexibility or altering the positions of elements at the decoding region.

Another reaction which is affected by the cleavage in 16S rRNA is translocation, which was 10 times faster and less sensitive to inhibition by viomycin on colicin E3-treated than on intact ribosomes. Translocation is catalysed by EF-G and entails the movement of the tRNA–mRNA complex through the ribosome. Our data suggest that colicin E3 cleavage accelerated the movement of tRNA through the ribosome, whereas the rate of Pi release from EF-G after GTP hydrolysis was not changed. On native ribosomes, the rates of Pi release and tRNA movement are limited to 30 s^−1^ by a ribosome rearrangement termed unlocking (Savelsbergh *et al*., 2003). In contrast, tRNA translocation on the cleaved ribosomes is faster (110 s^−1^) than unlocking (and translocation) on untreated ribosomes. This suggests that the cleavage of 16S rRNA leads to uncoupling of tRNA movement from unlocking. In other words, the step which usually limits the rate of tRNA movement during translocation appears to depend on the structure and/or dynamics of the decoding site.

The ribosomes cleaved by colicin E3 are capable of translocation even in the presence of viomycin, although at a lower rate than in the absence of the antibiotic, whereas on intact ribosomes, the translocation is completely abolished by the drug. There are two (non-exclusive) mechanisms by which viomycin may block translocation: it greatly stabilizes tRNA in the A site (Peske *et al*., 2004) and blocks ribosomes in the ratcheted state with tRNAs in their hybrid A/P and P/E positions (Ermolenko *et al*., 2007). Both mechanisms may lead to an inhibition of translocation by stabilizing, respectively, the ground state of the pre-translocation complex or an intermediate which is transient otherwise. However, both the stability of peptidyl-tRNA in the A site (Fig. 4E) and the ratcheting movements of the ribosome (see Discussion above) are not altered by colicin E3 cleavage, and it is therefore unlikely that the lowered sensitivity to viomycin is due to differences at these stages. Alternatively, viomycin may restrict the movements of the ribosome in a way which abolishes not only intersubunit movements, but also the flexibility within the 30S subunit. Cleavage of 16S rRNA may increase the mobility of the 30S subunit head and render the ribosomes flexible even in the presence of viomycin, thus allowing for slow but complete translocation in the presence of the antibiotic. While the structural consequences of the 16S rRNA cleavage remain speculative, the present data suggest that alterations at the decoding centre that affect the conformational flexibility are crucial for translocation and determine the rate of tRNA movement through the ribosome.

## Experimental procedures

### Buffers and reagents

All experiments were carried out in buffer A (50 mM Tris-HCl, pH 7.5, 70 mM NH_4_Cl, 30 mM KCl, 7 mM MgCl_2_) at 37°C. *In vitro* translation was performed in buffer B (50 mM Tris-HCl, pH 7.5, 70 mM NH_4_Cl, 30 mM KCl, 3 mM MgCl_2_). GTP, phosphoenolpyruvate and pyruvate kinase were from Roche Diagnostics. Radioactive compounds were from MP Biomedicals or Hartmann Analytics. All other chemicals were from Merck.

### Ribosomes, mRNAs, tRNAs and factors

70S ribosomes from *E. coli* MRE 600 were prepared as described (Rodnina and Wintermeyer, 1995). [^3^H]fMet-tRNA^fMet^, [^14^C]Phe-tRNA^Phe^, [^14^C]Val-tRNA^Val^, [^14^C]Tyr-tRNA^Tyr^, [^14^C]Ile-tRNA^Ile^ (all HPLC-purified), EF-Tu, EF-G, EF-Tu(H84A) and initiation factors were prepared as described (Rodnina *et al*., 1994a,b; 1997; 1999; Daviter *et al*., 2003). MFTI-mRNA with the sequence GGCAAGGAGGUAAAUAAUGUUCACGAUU where the start codon is underlined was purchased from Curevac (Germany). The mRNA used for the pentapeptide synthesis was 134 nt long, had the coding sequence indicated in Fig. 6A and was prepared by T7 RNA-polymerase transcription. The preparation of mRNA coding for 75 N-terminal amino acids of leader peptidase (Lep75 mRNA) is described elsewhere (Bornemann *et al*., 2008).

### Expression and purification of colicin E3 and immunity protein Im3

Colicin E3 rRNase was overexpressed as a complex with His-tagged immunity protein Im3. After purification of the complex by Ni-NTA affinity chromatography, colicin E3 was separated from Im3 by denaturation with 6 M guanidinium chloride where the Im3 remains bound to the Ni-NTA resin while the E3 rRNase is eluted. Isolated colicin E3 was refolded by dialysis against water, followed by dialysis against 50 mM potassium-phosphate (pH 7.0). Final purification was by gel filtration on a Superdex 75 column (Amersham-Pharmacia-Biotech) in the same buffer. Purified colicin E3 was dialysed against water, lyophilized and stored at −20°C (Walker *et al*., 2003). Im3 was extracted from the overexpression cell culture by sonication and the cell debris removed by centrifugation. Ammonium sulphate was added to the cell lysate up to 45% saturation. The supernatant was dialysed against 50 mM Tris-HCl pH 7.5, 1 mM EDTA, 1 mM DTT and 50 mM KCl. Im3 was purified further by anion exchange chromatography on DE-52 (50 ml column) using a gradient of 50 mM−1 M KCl in the same buffer. Gel filtration on Superdex 75 (Amersham-Pharmacia-Biotech), equilibrated with 50 mM potassium phosphate, pH 7, was used for final purification. Purified Im3 was lyophilized in 50 mM potassium phosphate, pH 7, and stored at −20°C.

### Analysis of colicin E3 cleavage of 16S rRNA

Ribosomes were incubated in buffer A with colicin E3 (for concentrations see text) at room temperature for 30 min. The cleavage reaction was stopped by the addition of Im3 followed by ethanol precipitation in 0.3 M sodium acetate, pH 5.2. After washing with 70% ethanol, the pellets were re-suspended in 3 M sodium acetate, pH 5.2, containing 0.5% SDS, and incubated for 10 min at 37°C. Proteins were removed by extracting the solution three times with water-saturated phenol followed by chloroform extraction. The rRNA was ethanol-precipitated, re-suspended in water and analysed by denaturing polyacrylamide gel electrophoresis.

### Biochemical assays

Ribosomes (1 μM) were incubated in buffer A with colicin E3 (1 μM) (E3-treated ribosomes) or without the rRNase (control ribosomes) for 30 min at room temperature. Im3 (1.1 μM) was added to each sample and incubated for another 5 min. These ribosomes were used for the biochemical assays.

Total translation was assayed as described (Buskiewicz *et al*., 2004). Ribosomes (0.5 μM) were mixed in buffer B with Lep75 mRNA (2 μM), initiation factors (0.85 μM), GTP (1 mM) and f[^3^H]Met-tRNA^fMet^ (1 μM) to form initiation complexes. Ternary complex was prepared in buffer B with putrescine (6.4 mM), phosphoenolpyruvate (3 mM), GTP (1 mM) and EF-Tu (50 μM) and incubated for 15 min at 37°C. Total aa-tRNA (21 μM) containing [^14^C]Leu-tRNA^Leu^ was added to EF-Tu, and the mixture was added to initiated ribosomes. After incubation at 37°C for various times, samples were removed and the amount of protein synthesized was determined by TCA precipitation and quantifying radioactivity.

Initiation complexes were formed by mixing 70S ribosomes (1 μM), [^3^H]fMet-tRNA^fMet^ (1.5 μM), mRNA (1.5 μM), GTP (1 mM) and initiation factors (1.5 μM each) and incubating for 1 h at 37°C. EF-Tu·GTP was prepared by adding GTP (1 mM), phosphenolpyruvate (3 mM) and pyruvate kinase (0.1 mg ml^−1^) to EF-Tu (0.9 μM) and incubating for 15 min at 37°C. Ternary complex was formed by the addition of [^14^C]Phe-tRNA^Phe^ (0.8 μM) and incubation for a further minute. Ternary complex (0.4 μM, unless stated otherwise) was added to initiation complexes (0.2 μM) and incubated at 37°C. At the indicated times, samples of ribosome complexes (10 pmol) were removed for analysis. The GTPase activity of EF-G and translocation under turnover conditions were measured as described (Savelsbergh *et al*., 2003; 2005), using catalytic amounts of EF-G (10 nM) and excess GTP (200 μM).

To synthesize pentapeptides, 70S ribosomes (1 μM) were initiated with mRNA coding for MetValTyrIlePhe (3 μM) as described above and [^14^C]Val–tRNA^Val^·GTP·EF-Tu, Tyr-tRNA^Tyr^·GTP·EF-Tu, Ile-tRNA^Ile^·GTP·EF-Tu, Phe-tRNA^Phe^·GTP·EF-Tu (1.1 μM each), EF-G (2 μM) and GTP (1 mM) were added; the reaction was incubated for 10 min at 37°C. A total of 10 pmol of ribosome complex was removed and analysed by nitrocellulose filtration or HPLC peptide analysis. The experiments were carried out in parallel with ^14^C-labelled Tyr, Ile or Phe instead of Val in the presence of the other four unlabelled aa-tRNAs.

### Rapid kinetics

Fluorescence stopped-flow measurements were performed and data analysed as described previously (Rodnina *et al*., 1997; Savelsbergh *et al*., 2003). For translocation experiments, pre-translocation complexes were prepared by mixing initiation and ternary complexes, resulting in ribosomes carrying deacylated tRNA^fMet^ in the P site and fMetPhe-tRNA^Phe^(Prf16/17) in the A site. Pre-translocation complexes (0.1 μM) were mixed rapidly with an equal volume of EF-G (2 μM after mixing) and GTP (1 mM) in buffer A at 37°C. Proflavin fluorescence was excited at 470 nm and measured after passing a cut-off filter (KV500, Schott).

Pi release from EF-G and EF-Tu was measured using the fluorescence change of phosphate-binding protein labelled with MDCC (2.5 μM) (Brune *et al*., 1994; Savelsbergh *et al*., 2003). For EF-G, equal volumes of pre-translocation complex (0.1 μM final) and EF-G (2 μM) with GTP (200 μM) in buffer A were mixed at 37°C. In the case of EF-Tu, equal volumes of initiation complex (0.1 μM final concentration) and EF-Tu·GTP·Phe-tRNA^Phe^ (2 μM final concentration) in buffer A were mixed in buffer A at 37°C. MDCC fluorescence was excited at 425 nm and measured after passing a cut-off filter (KV450, Schott).

Kinetics of peptide bond formation were measured by quench-flow using a Kintek RQF-3 apparatus. Purified 70S initiation complexes (0.4 μM) were mixed with a threefold excess of ternary complex, then the reaction was quenched by 0.8 M KOH (Gromadski and Rodnina, 2004b). Samples were hydrolysed for 30 min at 37°C and neutralized with acetic acid. The amount of fMetPhe formed was determined by reversed-phase HPLC (Lichrospher 100 RP-8, Merck). The rate of translocation was determined by the reaction of translocated peptidyl-tRNA with Pmn, as monitored by quench-flow (Katunin *et al*., 2002a). Pre-translocation complexes (0.25 μM) were mixed with EF-G (3 μM) and Pmn (10 mM); the reactions were stopped and samples treated as described above. The amount of f[^3^H]Met[^14^C]Phe-Pmn was analysed by reversed-phase HPLC.
